# Supplementary material for: Drug repurposing for aging research using model organisms
Source: Aging Cell. 2017 Jun 16;16(5):1006–15. doi: 10.1111/acel.12626 (PMC5595691; doi:10.1111/acel.12626)
Supplement: Supplementary file 7 — Data S1 Zip‐Archive of all report cards. [file ACEL-16-1006-s007.zip › RC_MIX.pdf]

MIX

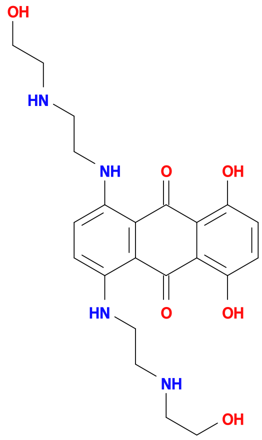

Database identifiers

ChEMBLCompound	CHEMBL58
DrugBank	DB01204
CHEBI	50729
eMolecules	742146

Ranking

	Rank	Score
Drosophila	397/697	0.365
C. elegans	287/591	0.199

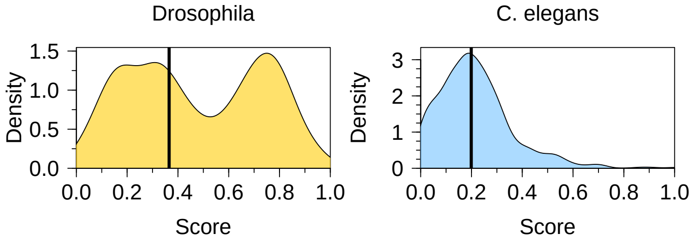

	Ageing implication	Domain conservation	Binding site conservation	Binding affinity	Bioavailability	Lipinski	Promiscuity	Purchasability	Drug approval	**Total**
Drosophila	0.624	0.963	0.883	0.452	(0.9)	-0.05	-0.0	0.1	0.1	0.365
C. elegans	0.624	0.963	0.847	0.452	0.216	-0.05	-0.0	0.1	0.1	0.199

Names

- **mitoxantrone**
- 1,4-dihydroxy-5,8-bis(2-(2-hydroxyethyl)aminoethylamino)-9,10-anthracenedione
- Novantrone

Roles

analgesic, antineoplastic agent

Status

Approved drug (according to ChEMBL)	Yes
Administration Route	Intravenous
Number of Rule of 5 violations	1
Binding affinity to original target in log units (RF-Score prediction)	4.81
Burns *C. elegans* bioavailability prediction	-4.8

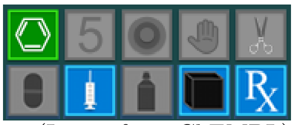

(Image from ChEMBL)

Medical Information

Indicatation: For the treatment of secondary (chronic) progressive, progressive relapsing, or worsening relapsing-remitting multiple sclerosis
Mechanism of action: Mitoxantrone, a DNA-reactive agent that intercalates into deoxyribonucleic acid (DNA) through hydrogen bonding, causes crosslinks and strand breaks. Mitoxantrone also interferes with ribonucleic acid (RNA) and is a potent inhibitor of topoisomerase II, an enzyme responsible for uncoiling and repairing damaged DNA. It has a cytocidal effect on both proliferating and nonproliferating cultured human cells, suggesting lack of cell cycle phase specificity.
Toxicity: Severe leukopenia with infection.
Metabolism: Hepatic
Retail: APP Pharmaceuticals, Bedford Labs, Ben Venue Laboratories Inc., EMD Canada Inc., Hospira Inc., Otn Generics Inc. and 5 more suppliers
Mitoxantrone 25 mg/12.5 ml vial - 37.5 USD / ml
Mitoxantrone 20 mg/10 ml vial - 42.0 USD / ml
Novantrone 2 mg/ml vial - 158.59 USD / ml
Novantrone 2 mg/ml Concentrate 10ml Vial - 1649.32 USD / vial

(Information from DrugBank)

Compound Target Characteristics

DNA topoisomerase 2-beta

Best gene implication in ageing for this target family came from gene F1M7A0 via mapping the annotation from RGD 62048 annotated in RGD 2014-03-11. Annotation GO 7568 (aging) was Inferred from Expression Pattern

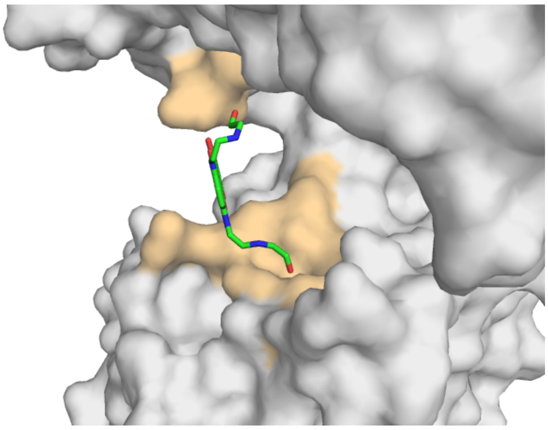

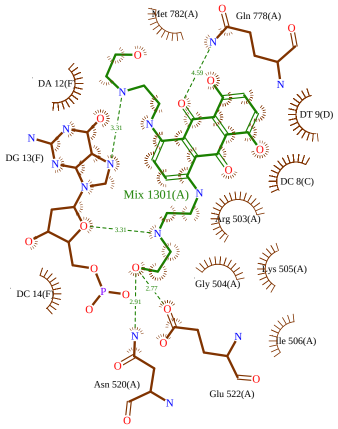

protein	amino acids contacts (binding site)
PDB:4g0v:chainA:Q02880	R G K I L N E Q M
tr:E9PCY5:E9PCY5_HUMAN	R G K I L N E Q M
sp:Q02880:TOP2B_HUMAN	R G K I L N E Q M
tr:F1M7A0:F1M7A0_RAT	R G K I L N E M M
sp:Q64511:TOP2B_MOUSE	R G K I L N E Q M
tr:Q6NR40:Q6NR40_DROME	R G K L L N E V M
sp:P15348:TOP2_DROME	R G K L L N E V M
sp:Q23670:TOP2_CAEEL	R G K L L N E Q G
tr:E9P8H0:E9P8H0_YEASX	R G K M L N E Q Q
tr:E9P8H2:E9P8H2_YEASX	R G K M L N E Q Q
tr:E9P8H4:E9P8H4_YEASX	R G K M L N E Q Q
tr:E9P8H5:E9P8H5_YEASX	R G K M L N E Q Q
tr:E9P8H7:E9P8H7_YEASX	R G K M L N E Q Q
tr:E9P8H8:E9P8H8_YEASX	R G K M L N E Q Q
tr:Q7LMA0:Q7LMA0_YEASX	R G K M L N E Q Q
tr:E9P8G9:E9P8G9_YEASX	R G K M L N E Q Q
sp:P06786:TOP2_YEAST	R G K M L N E Q Q

	whole protein		domain-based		contact-based	
protein	ident	simil	ident	simil	ident	simil
PDB:4g0v:chainA:Q02880	1.0	1.0	1.0	1.0	1.0	1.0
tr:E9PCY5:E9PCY5_HUMAN	0.71	0.71	1.0	1.0	1.0	1.0
sp:Q02880:TOP2B_HUMAN	1.0	1.0	1.0	1.0	1.0	1.0
tr:F1M7A0:F1M7A0_RAT	0.64	0.83	0.87	0.96	0.89	0.88
sp:Q64511:TOP2B_MOUSE	0.96	0.98	0.99	1.0	1.0	1.0
tr:Q6NR40:Q6NR40_DROME	0.41	0.58	0.75	0.93	0.78	0.88
sp:P15348:TOP2_DROME	0.47	0.71	0.75	0.93	0.78	0.88
sp:Q23670:TOP2_CAEEL	0.45	0.73	0.73	0.92	0.78	0.85
tr:E9P8H0:E9P8H0_YEASX	0.34	0.64	0.58	0.86	0.78	0.87
tr:E9P8H2:E9P8H2_YEASX	0.34	0.64	0.58	0.86	0.78	0.87
tr:E9P8H4:E9P8H4_YEASX	0.34	0.64	0.58	0.86	0.78	0.87
tr:E9P8H5:E9P8H5_YEASX	0.34	0.64	0.58	0.86	0.78	0.87
tr:E9P8H7:E9P8H7_YEASX	0.34	0.64	0.58	0.86	0.78	0.87
tr:E9P8H8:E9P8H8_YEASX	0.34	0.64	0.58	0.86	0.78	0.87
tr:Q7LMA0:Q7LMA0_YEASX	0.34	0.64	0.58	0.86	0.78	0.87
tr:E9P8G9:E9P8G9_YEASX	0.34	0.64	0.58	0.86	0.78	0.87
sp:P06786:TOP2_YEAST	0.34	0.64	0.58	0.86	0.78	0.87

Top2 (FBgn0003732) associated phenotypes

developmental rate defective, heat sensitive, lethal - all die before end of first instar larval stage, lethal - all die before end of second instar larval stage, lethal - all die during first instar larval stage, male fertile, neuroanatomy defective, partially lethal - majority die, small body, somatic clone

(Information from FlyBase)

Top2 (UniProt:Q6NR40) annotation

Function: Control of topological states of DNA by transient breakage and subsequent rejoining of DNA strands. Topoisomerase II makes double-strand breaks. (RuleBase:RU000381).
Subunit: Homodimer. ECO:0000256—RuleBase:RU000381.

(Information from UniProt)

Top2 (UniProt:P15348) annotation

Function: Control of topological states of DNA by transient breakage and subsequent rejoining of DNA strands. Topoisomerase II makes double-strand breaks.
Cofactor: Mg(2+) Evidence=(PROSITE-ProRule:PRU00995); Name=Mn(2+); Xref=ChEBI:CHEBI:29035; Evidence=(PROSITE-ProRule:PRU00995); Name=Ca(2+); Xref=ChEBI:CHEBI:29108; Evidence=(PROSITE-ProRule:PRU00995); Note=Binds two Mg(2+) per subunit. The magnesium ions form salt bridges with both the protein and the DNA. Can also accept other divalent metal cations, such as Mn(2+)

or Ca(2+). (PROSITE-ProRule:PRU00995);
Subunit: Homodimer.
Subcellular location: Nucleus.
Miscellaneous: Eukaryotic topoisomerase I and II can relax both negative and positive supercoils, whereas prokaryotic enzymes relax only negative supercoils.

(Information from UniProt)

K12D12.1 (UniProt:Q23670) annotation
Function: Control of topological states of DNA by transient breakage and subsequent rejoining of DNA strands. Topoisomerase II makes double-strand breaks (By similarity).
Cofactor: Mg(2+) Evidence=(PROSITE-ProRule:PRU00995); Name=Mn(2+); Xref=ChEBI:CHEBI:29035; Evidence=(PROSITE-ProRule:PRU00995); Name=Ca(2+); Xref=ChEBI:CHEBI:29108; Evidence=(PROSITE-ProRule:PRU00995); Note=Binds two Mg(2+) per subunit. The magnesium ions form salt bridges with both the protein and the DNA. Can also accept other divalent metal cations, such as Mn(2+) or Ca(2+). (PROSITE-ProRule:PRU00995);
Subunit: Homodimer.
Subcellular location: Nucleus
Miscellaneous: Eukaryotic topoisomerase I and II can relax both negative and positive supercoils, whereas prokaryotic enzymes relax only negative supercoils.

(Information from UniProt)
